# Comparison of the ALKA and CRS scores to predict outcomes among patients with coronavirus disease 2019 infection in United Arab Emirates

**DOI:** 10.3389/fmed.2025.1553189

**Published:** 2025-08-18

**Authors:** Bushra Kurban, Adnan Agha, Lutfi Ali S. Kurban, Virgie Guy, Javed Yasin, Fayez Alshamsi, Mohamed Ismail, Omran Bakoush

**Affiliations:** ^1^Department of Internal Medicine, College of Medicine and Health Sciences, United Arab Emirates University, Al Ain, United Arab Emirates; ^2^Department of Radiology, College of Medicine and Health Sciences, United Arab Emirates University, Al Ain, United Arab Emirates

**Keywords:** clinical decision, comorbidity, COVID-19, critical illness, hospitalization, mortality, outpatient, risk assessment

## Abstract

**Introduction:**

The coronavirus disease 2019 (COVID-19) pandemic resulted in significant global mortality and morbidity, with emerging mutant strains continuing to potentially precipitate severe respiratory illness. Two clinical assessment tools, namely, the COVID-19 Risk of Complications Score (CRS), based on 13 comorbidities, and the ALKA (age, lactate dehydrogenase, kidney function, and albumin) score have been developed to predict disease severity among patients who are symptomatic at presentation. This study aimed to compare the performance of these two risk-scoring systems in predicting hospital admission, critical illness, and mortality.

**Methods:**

This retrospective study included 368 patients diagnosed with COVID-19 at SEHA hospitals in Al Ain over a six-month period. The CRS and ALKA scores were calculated to predict hospital admission, critical illness, and mortality. Predictive ability was assessed using receiver operating characteristic (ROC) curve analysis. Odds ratios (ORs) were calculated to assess the risk of hospital admission, critical illness, and mortality.

**Results:**

The mean age of the patients was 51 ± 19.42 years, and 145 (39.4%) of them were male. Among the patients, 162 required inpatient care, 13 required invasive ventilation, and the mortality rate was 4.9% (eight patients). ROC analysis revealed that ALKA outperformed CRS in predicting hospital admission (ALKA area under the curve [AUC] 0.79 vs. CRS AUC 0.71), critical illness (ALKA AUC 0.76 vs. CRS AUC 0.67), and mortality (ALKA AUC 0.96 vs. CRS AUC 0.82). The OR for ALKA outperformed CRS in predicting hospital admission (ALKA 3.12 vs. CRS 1.12), critical illness (ALKA 2.9 vs. CRS 2.01), and mortality (ALKA 6.25 vs. CRS 1.1).

**Conclusion:**

Our study demonstrated that ALKA score outperforms CRS in predicting hospital admission, critical illness, and mortality among patients with symptomatic COVID-19 at initial presentation. Further external validation of both tools is required to assess their effectiveness in different healthcare settings.

## 1 Introduction

The novel coronavirus disease 2019 (COVID-19) emerged in Wuhan, China, in December 2019 and rapidly spread worldwide (1). During the 2020–2021 pandemic, COVID-19 became the third leading cause of death following cardiovascular disease and cancer (2). Despite widespread vaccination, COVID-19 continues to persist as an endemic disease, with small recurrent outbreaks that may lead to severe complications (3). Although COVID-19 infections are typically mild, the risk of severe illness and death is significantly higher in older adults and those with comorbidities, such as hypertension, diabetes, and chronic obstructive pulmonary disease (COPD) (4).

Although many risk assessment tools are employed in clinical practice to triage patients with COVID-19 infection at initial assessment, the COVID-19 Risk of Complications Score (CRS) is one of the most established risk assessment tools in the United States of America (USA) (5). The CRS is calculated based on patients’ demographic and multiple clinical risk factors (6). Despite its promise in predicting complications, CRS has several limitations. First, it incorporates several different morbidity risk factors, rendering it potentially cumbersome to apply in routine clinical practice during the initial assessment, especially in resource-limited contexts where the electronic health record (EHR) system may not be readily available or fully integrated. Second, this score has limited generalisability due to the lack of external validation, which restricts its applicability across diverse patient populations and settings (7).

Point-of-care assessment tools play a crucial role in guiding patient management decisions (8). Therefore, the importance of a simple, valid, and practical clinical assessment tool at the time of diagnosis cannot be overstated. Such a tool is critical for effective patient triage, timely initiation of supportive and therapeutic care, and the optimal use of healthcare resources.

Our group has developed the ALKA score, a simple tool to predict COVID-19 complications, using four variables: age, lactate dehydrogenase (LDH), kidney function, and albumin (9). Unlike other risk assessment tools that rely on potentially inaccurate clinical histories or comorbidities, age is a clinical parameter that is both readily available and reliable as a predictor of disease severity. This tool utilises these four variables to categorise patients into three risk categories based on their likelihood of progressing to a critical illness. This study aimed to validate the performance of the ALKA score and CRS in risk-stratifying patients with COVID-19 at the initial diagnosis. Specifically, we assessed the diagnostic accuracy of both scoring systems in predicting key patient outcomes, need for hospital admission, and risk of progression to critical illness and death.

## 2 Materials and methods

### 2.1 Participants and study design

**Figure 1:**
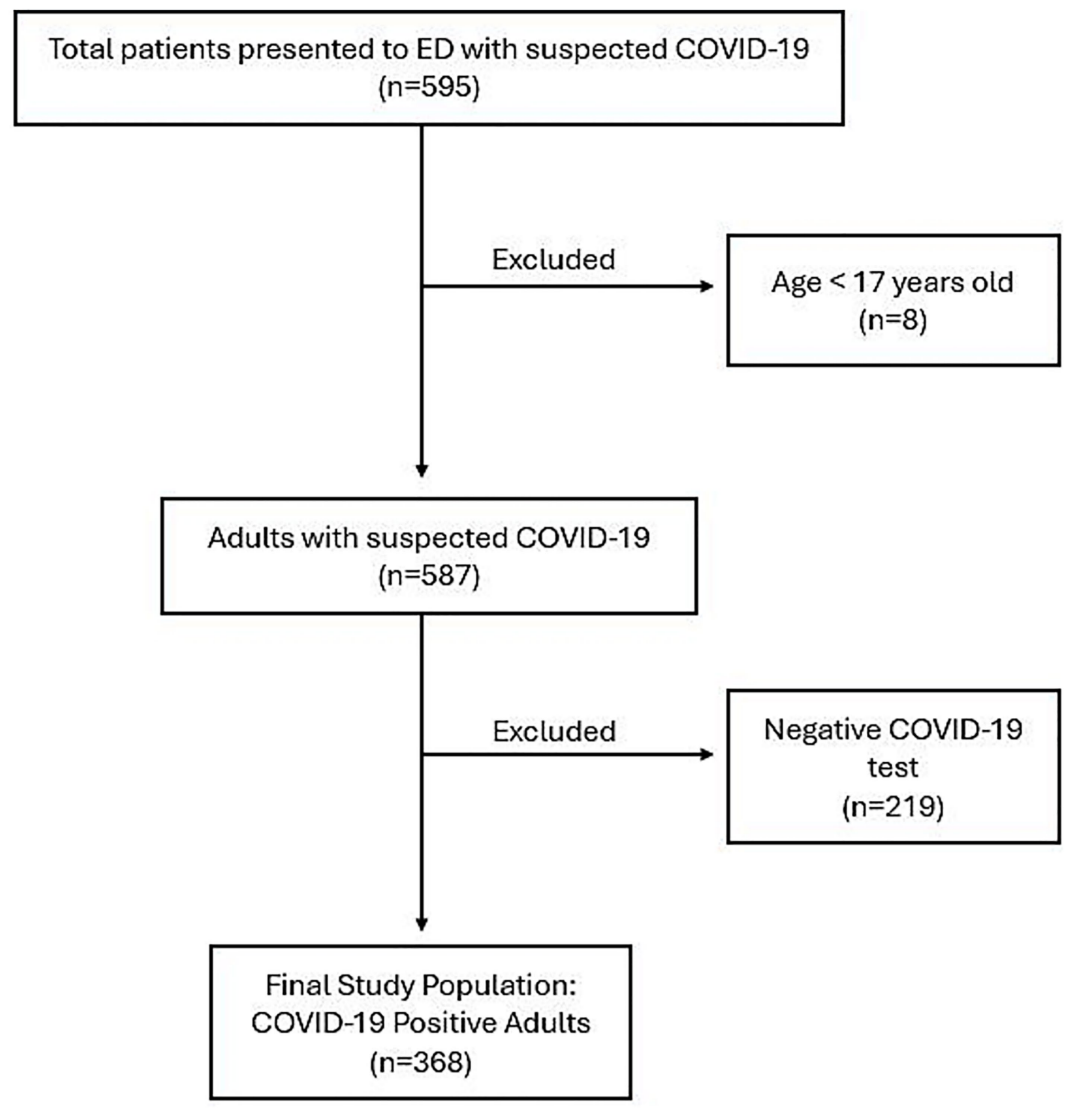
Flowchart of patient selection. ED, Emergency Department.

A consecutive sampling method was used at SEHA hospitals (Tawam Hospital and Al Ain Hospital), the two primary hospitals in Al Ain city, Abu Dhabi, United Arab Emirates, designated for treating COVID-19 patients. A total of 595 patients with suspected COVID-19 symptoms presented to the Emergency Departments (EDs) of these two hospitals between November 27, 2022, and May 24, 2023. As illustrated in Figure 1, eight patients under the age of 17 were excluded. Among the remaining patients, 368 adults (145 males and 223 females) were confirmed to have COVID-19 and were included in the final analysis. As these hospitals were the sole COVID-19 treatment centres in the region, all patients were directly referred to these two facilities. Confirmation of positive COVID-19 results was based on real-time reverse transcription-polymerase chain reaction assays of nasopharyngeal swab specimens (AllplexTM 2019-nCoV Assay, Seegene Inc., Seoul, Republic of Korea).

The decision to admit the patient was based on the clinical judgement of the treating physicians based on the severity of clinical illness using a combination of clinical and radiological evaluations along with CRS, as outlined by Halalau et al. (5). The ALKA score was applied retrospectively and did not influence clinical decision-making at the time. The data for this study was collected by a different group of investigators than those who originally developed the ALKA score. This approach supports evaluation in a real-world clinical context and helps reduce the potential for investigator bias, offering a more independent assessment of the score’s performance. In accordance with the Abu Dhabi Department of Health guidelines, all high-risk patients were treated with sotrovimab (monoclonal antibodies) irrespective of their symptoms. Additionally, patients who were symptomatically high-risk, had low oxygen saturation, and had radiological evidence of pneumonia received antiviral therapy in combination with sotrovimab (10).

### 2.2 Data collection

Data from the EHR were collected using a standardised data collection tool. Patient demographics, medical history, laboratory biomarkers, and outcomes were documented and analysed. At initial presentation to the Emergency Department, blood samples were collected from all patients with suspected COVID-19 in accordance with hospital protocol. The blood samples were routinely analysed for key laboratory parameters, including serum LDH, serum albumin, and eGFR. Best practices to minimise the missing data were followed during data collection and the blood samples were stored in the hospital laboratory for potential future retesting for the missing laboratory data (11). This process ensured a complete dataset for all included patients, and therefore a complete dataset was obtained for all recruited patients, and the analysis was conducted on complete dataset.

Data on ventilatory support requirements, admission to intensive care unit (ICU), discharge, and death were recorded. Comorbidities were identified based on the medical history, previous visits or at the time of presentation. Obesity was defined as a body mass index ≥ 30 and was calculated as weight in kilograms divided by height in meters squared. The data were stored in a password-protected file, and the identities of patients were kept anonymous.

### 2.3 Ethical approval

This study was approved by the Department of Health Ethical Committee and the requirement for written informed consent was waived (DOH/CVDC/2022/1464).

### 2.4 Calculation of risk scores

#### 2.4.1 CRS

CRS is scored based on age, sex, and presence of comorbidities, including coronary artery disease, congenital heart disease, congestive heart failure, end-stage renal disease, end-stage liver disease, COPD, diabetes, hypertension, obesity, pregnancy status, and immunocompromised status. The maximum score is 15, and each of the 13 reported items receives 1 point if present, apart from age, at which a patient may receive 0–3 points. Thereafter, the total score is categorised into the following three risk groups: low (green, score 0–2), intermediate (yellow, score 3–5), and high (red, score 6–15).

#### 2.4.2 ALKA

As elucidated by Kurban et al. (9) the ALKA score was calculated by incorporating the patients’ age, serum LDH, serum albumin, and eGFR. Based on the critical illness probability, the score was divided into the following three categories: low (less than 5% risk), moderate (5–10%), and high (greater than 10%). Of note, the ALKA score does not require a detailed medical history or a comprehensive list of comorbidities, making it a practical tool for early risk assessment.

### 2.5 Outcomes

The primary outcome of interest was progression to critical illness. The secondary outcomes were the need for hospital admission and all-cause mortality. Critical illness was defined based on the Chinese management guidelines for COVID-19 as a severe condition encompassing outcomes such as death, septic shock, or respiratory failure requiring invasive or non-invasive ventilatory support (9, 12).

### 2.6 Statistical analysis

With an expected disease prevalence of 5%, C-statistic of 0.8, and 95% confidence interval, the required sample size was estimated to be 308 patients. Additionally, a similar sample size has been deemed adequate for external validation of prognostic models predicting adverse outcomes in COVID-19 patients, as supported by a recently published meta-analysis (13–16).

The quantitative variables were reported as medians and ranges. Categorical variables were presented as frequencies and proportions. Discrimination was evaluated using C-statistics, along with the corresponding 95% confidence intervals and receiver operating characteristic (ROC) curve. AUC was computed using the trapezoidal rule, a numerical integration method, in SPSS programme. C-statistics below 0.6, between 0.6–0.7, above 0.7, and above 0.8 were considered to represent poor, moderate, good, and excellent quality, respectively. Crosstabs were generated for each risk stratification score by comparing the predicted risk categories (low, moderate, and high) with observed clinical outcomes, including hospital admission, critical illness, and mortality. The Youden index was used to determine the optimal cut-off point that maximised both sensitivity and specificity. Statistical comparison of the AUCs was performed using MedCalc software and the DeLong test (17). We evaluated the calibration of the ALKA and CRS models for predicting the three outcomes using the Hosmer-Lemeshow test. Model fit was assessed with the likelihood ratio, chi-square (χ^2^) statistic, and explained variance was measured by Nagelkerke’s R^2^.

The Pearson Chi-Square test was used to determine whether a significant association existed between the risk categories and outcomes. To adjust for multiple comparisons, a Bonferroni correction was applied. Bonferroni-adjusted standardized residuals were calculated to evaluate significance across outcome groups. Additionally, the likelihood ratio was included to further assess potential linear trends across the risk groups. To provide a more comprehensive evaluation of the relationship between the risk scores and clinical outcomes, the odds ratio (OR) was calculated using univariate logistic regression analysis for each outcome, allowing for an assessment of the strength of the association between the risk categories and the likelihood of adverse events. We performed a *post hoc* power analysis for mortality outcome by grouping ALKA and CRS scores into high-risk versus non-high risk (which included low and intermediate risk groups). Power calculations were done using the online tool ClinCalc with an alpha level of 0.05 (18). *p*-values < 0.05 were considered as significant. All analyses were performed using the IBM SPSS software (version 27, Chicago, IL, USA).

## 3 Results

This study included 368 patients, with a mean age of 51 ± 19.42 years. The study cohort comprised 145 males and 223 females. Of these, 202 patients did not require hospital admission, whereas 162 were hospitalised. Among those who were hospitalised, six were transferred to the ICU. The mean hospital stay was 6 ± 6.7 days for patients admitted to the general ward and 14.6 ± 19.6 days for those admitted to the ICU. Furthermore, 13 patients required invasive ventilation, and eight deaths were recorded.

Of the 368 patients, 122 (33.1%) patients had diabetes, 140 (38.0%) had hypertension, 66 (17.9%) had asthma or COPD, 23 (6.3%) had congestive heart failure, 15 (4.1%) had coronary artery disease, 13 (3.5%) had chronic kidney disease, 13 (3.5%) had end-stage renal disease, 2 (0.5%) had liver disease, 4 (1.1%) were immunocompromised, and 5 (1.4%) were pregnant. Overall, 164 patients were classified as high-risk according to the Department of Health guidelines and received sotrovimab treatment. Of these, 4 patients developed critical illness, and 1 patient died. In the subgroup of 63 patients without comorbid risk factors, 10 patients were admitted to the hospital. However, none of the patients developed critical illness or died.

### 3.1 Diagnostic accuracy of CRS and ALKA for predicting hospital admission, critical illness, and mortality

The diagnostic performance of CRS and ALKA scores in predicting key clinical outcomes, namely, hospital admission, critical illness, and death were assessed using ROC curve analysis.

**Figure 2:**
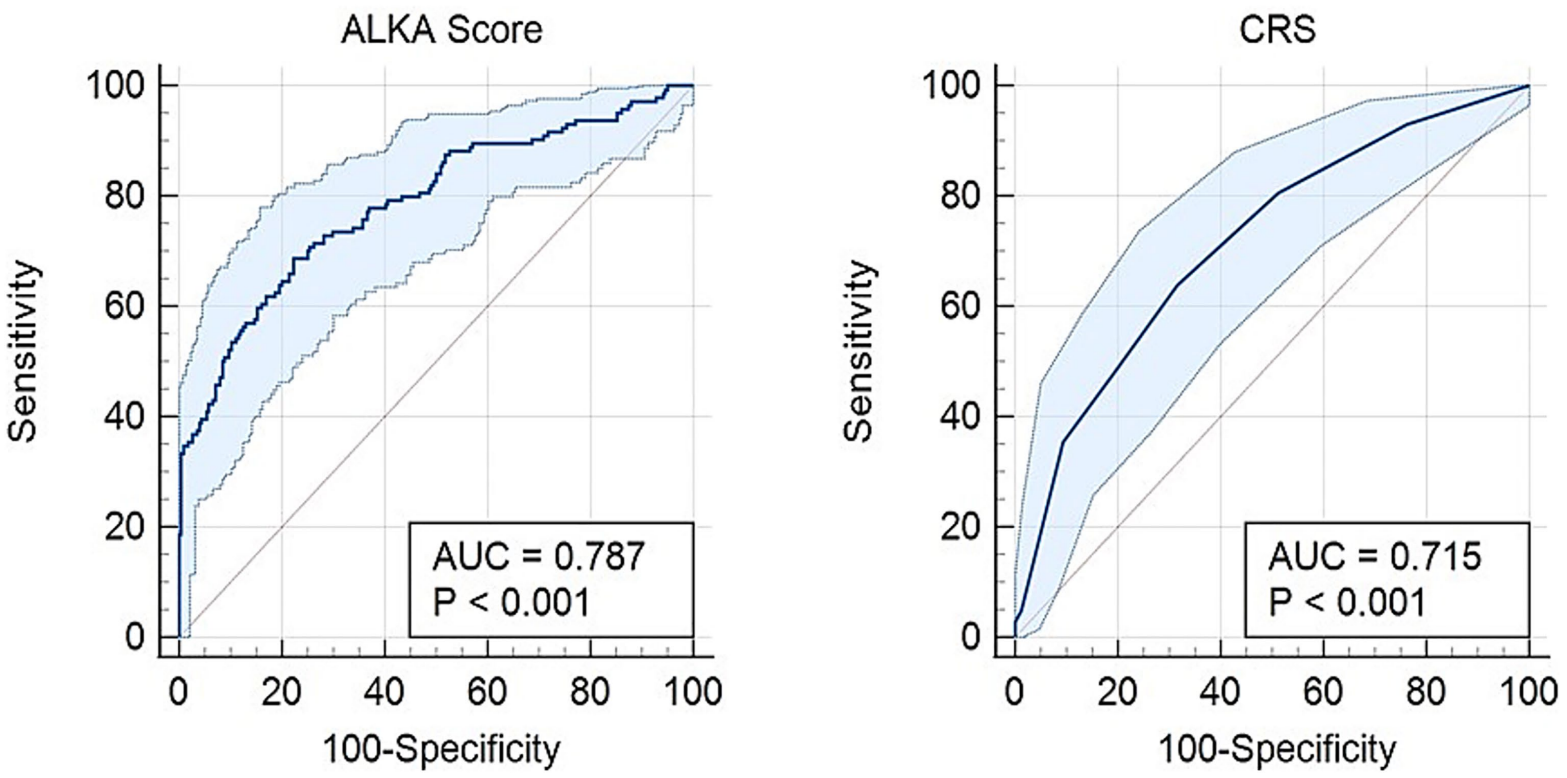
Receiver operating characteristic (ROC) curve comparing the predictive performance of the ALKA score and CRS for hospital admission in COVID-19 patients. The ALKA score demonstrated a higher area under the curve (AUC) of 0.79 (95% CI: 0.74–0.84), indicating superior predictive accuracy, whereas the CRS showed a lower AUC of 0.71 (95% CI: 0.66–0.77).

For hospital admission, as shown in Figure 2, ALKA score had an area under the curve (AUC) of 0.79 (95% CI: 0.74–0.84), while CRS had an AUC of 0.71 (95% CI: 0.66–0.77). The difference between the two AUCs was 0.0723 (95% CI, 0.029–0.115) with a *p*-value < 0.01. An ALKA cut-off point of 7.7%, at a maximum Youden index of 0.45, had a sensitivity of 71% and specificity of 75% in predicting admission, with a false positive rate (FPR) of 25% and a false negative rate (FNR) of 29%. A CRS cut-off point of 2.5, at a maximum Youden index of 0.32, showed a sensitivity of 64% and specificity of 68% for admission prediction, with a FPR of 32% and an FNR of 36%. Calibration analysis demonstrated that both ALKA and CRS performed well for prediction of admission, with Hosmer-Lemeshow *p* = 0.190 and 0.723, respectively. ALKA had stronger model fit (χ^2^ = 114.2, *p* < 0.01 vs. 52.6, p < 0.01) and explained more variance (Nagelkerke R^2^ = 0.362 vs. 0.181).

**Figure 3:**
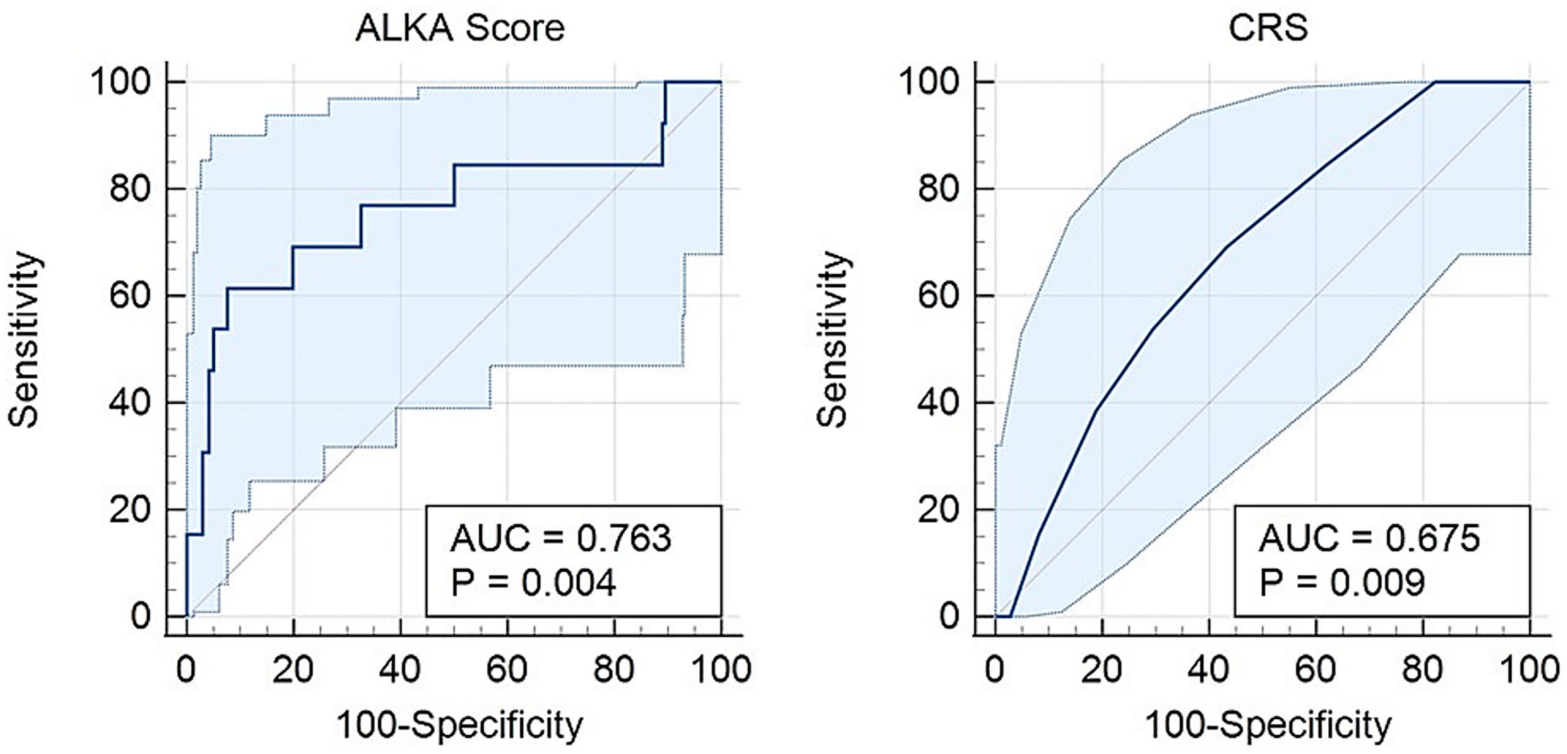
Receiver operating characteristic (ROC) curve comparing the CRS and ALKA scores in predicting critical illness in COVID-19 patients. The ALKA score demonstrated a higher area under the curve (AUC) of 0.763 (95% CI: 0.59–0.93) compared to CRS with an AUC of 0.675 (95% CI: 0.54–0.80).

Regarding critical illness, Figure 3 presents an AUC of 0.76 (95% CI: 0.59–0.93) for the ALKA score, while the CRS exhibits an AUC of 0.67 (95% CI: 0.54–0.80). The difference between the two AUCs was 0.0875 (95% CI: −0.051-0.226) with a *p*-value = 0.21. An ALKA cut-off point of 18.3%, corresponding to a maximum Youden index of 0.49, demonstrated a sensitivity of 69% and a specificity of 80% in predicting critical illness, and resulted in a false positive rate (FPR) of 20% and a false negative rate (FNR) of 31%. In comparison, a CRS cut-off point at its maximum Youden index of 0.26 attained a sensitivity of 69% and a specificity of 57%, with an FPR of 43% and an FNR of 31%. Both models showed good calibration for predicting critical illness, with Hosmer-Lemeshow *p*-values of 0.468 and 0.903, and chi-square statistics of 9.176 (*p* < 0.01) and 3.721 (*p* = 0.054), respectively. ALKA showed better discriminative performance with a model chi-square of 9.176 (*p* < 0.01) versus 3.721 (*p* = 0.054) for CRS. Variance explained was higher for ALKA (Nagelkerke R^2^ = 0.094) compared to CRS (0.038).

**Figure 4:**
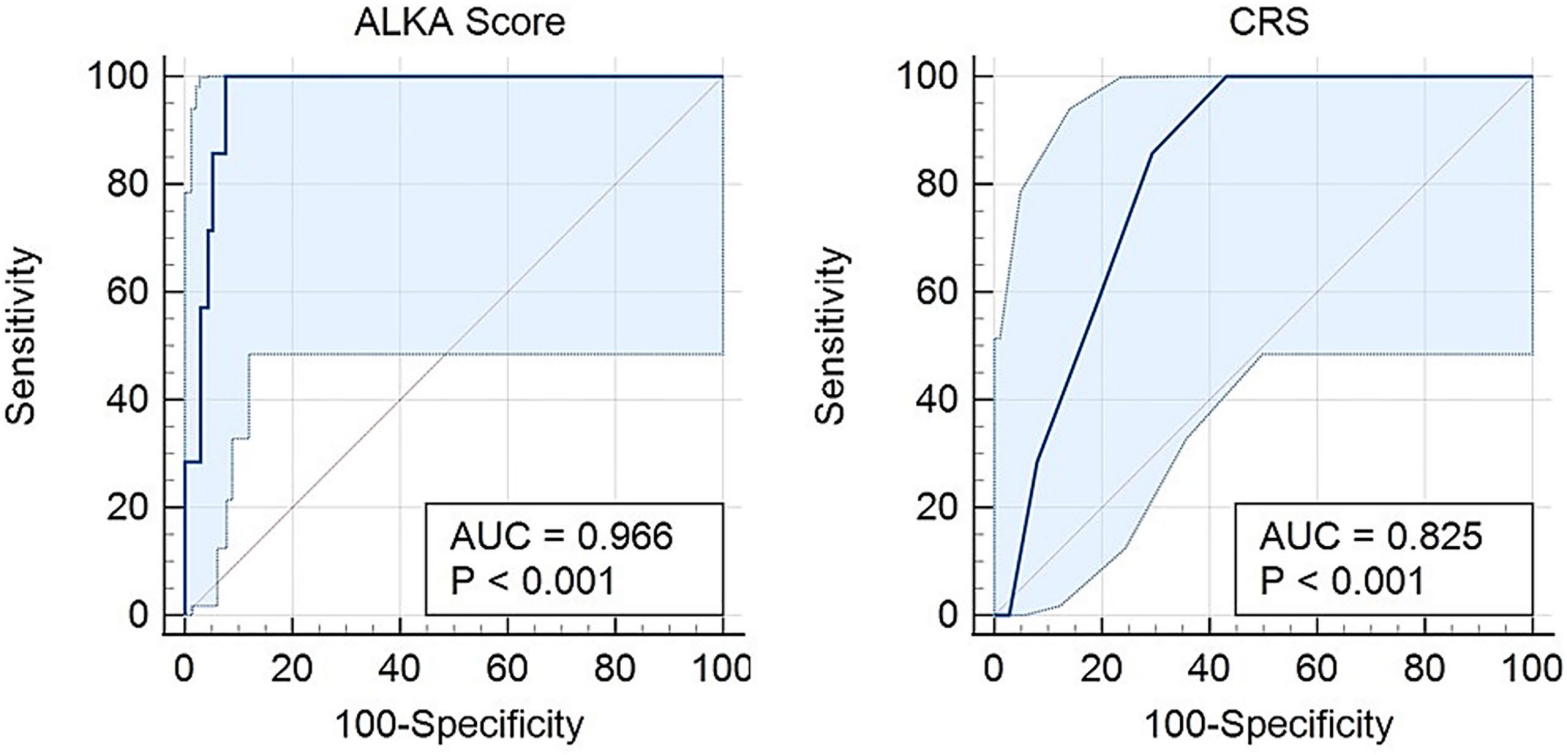
Receiver operating characteristic (ROC) curves for the prediction of death in COVID-19 patients using the CRS and ALKA scores. The ALKA score demonstrated excellent predictive performance with an area under the curve (AUC) of 0.966 (95% CI: 0.94–0.99). By comparison, the CRS showed lower predictive accuracy with an AUC of 0.82 (95% CI: 0.74–0.91).

For death outcomes, ALKA score achieved an AUC of 0.96 (95% CI: 0.94–0.99), while CRS demonstrated an AUC of 0.82 (95% CI: 0.74–0.91) (Figure 4). The difference between the two AUCs was 0.141 (95% CI: 0.053–0.229) with a *p*-value < 0.01. With a maximum Youden index of 0.92, ALKA cut-off point of 36.2% achieved a sensitivity of 100% and a specificity of 92% for predicting mortality, leading to a false positive rate (FPR) of 8% and a false negative rate (FNR) of 0%. In contrast, a CRS cut-off point of 3.5, at its peak Youden index of 0.56, demonstrated a sensitivity of 86% and a specificity of 71%, with an FPR of 29% and an FNR of 14%. Both ALKA and CRS models were well calibrated for predicting death (Hosmer-Lemeshow *p* = 0.996 and 0.726, respectively). ALKA had stronger model fit (χ^2^ = 32.210, *p* < 0.01 vs. 7.619, *p* < 0.01) and explained more variance (Nagelkerke R^2^ = 0.488 vs. 0.119).

ALKA outperformed CRS in predicting both hospital admission and in-hospital mortality. The difference in AUC between the two models was 0.072 for admission and 0.141 for mortality, both of which were statistically significant (*p* < 0.01). This indicates that ALKA provided a modest but meaningful improvement in discriminative ability for admission and a more substantial improvement for predicting death. No significant difference was observed between the models for predicting critical illness.

### 3.2 Distribution of patient outcomes by risk category

The patients were classified into three risk categories (low, intermediate, and high) based on their CRS and ALKA scores. Table 1 summarises the distribution of hospital admission, critical illness, and death outcomes for each risk group.

**Table 1:** The table presents the number of events (admission, critical illness and death) for each risk category (low, intermediate, and high) in CRS and ALKA scoring systems.

Risk category	Total cases	Admissions	Critical illness	Deceased	Alive
CRS scoring system					
Green: Low Risk	203	52	4	0	203
Yellow: Intermediate Risk	135	70	7	5	130
Red: High Risk	30	22	2	2	28
Odds Ratio (95% CI)	1.12 (1.08–1.15)	2.01 (0.92–4.36)	1.1 (1.05–1.15)		
*p-*value*	0.00038	1.90, ns	0.27, ns		
ALKA scoring system					
Green: Low Risk	169	31	2	0	169
Yellow: Intermediate Risk	69	24	1	0	69
Red: High Risk	130	89	10	7	123
Odds Ratio (95% CI)	3.12 (2.38–4.09)	2.90 (1.31–6.43)	6.25 (1.11–35.25)		
*p*-value*	<0.0001	0.00825	0.0019		

*Bonferroni adjust p value, ns = non-significant.

For CRS score, among the 203 patients in the low-risk (green) group, only 4 patients (1.97%) developed critical illness, and no patients died. In the intermediate-risk (yellow) group (135 patients), 7 patients (5.19%) progressed to critical illness, and 5 patients died. In the high-risk (red) group (30 patients), 2 patients (6.67%) developed critical illness, and both patients died.

In the ALKA score system, the low-risk group included 169 patients, with only 2 (1.18%) progressed to critical illness and there were no deaths. In the intermediate-risk group (69 patients), 1 patient (1.45%) developed critical illness, but no deaths were reported. Among the 130 high-risk patients, 10 (7.69%) developed critical illness, and 7 of them died.

Notably, of the 13 patients who developed critical illness, 77% were classified as severe by the ALKA score, compared with only 15% by the CRS. Additionally, all 7 patients who died were classified as severe at presentation by the ALKA score, whereas only 27% were categorised as severe by the CRS.

### 3.3 Statistical associations between risk scores and outcomes

The relationships between the CRS and ALKA scores and key clinical outcomes, namely, hospital admission, critical illness, and deaths, were examined using Pearson’s chi-square and likelihood ratio tests. The results are summarised below, with the corresponding ORs for each outcome offering additional insights into the strength of these associations, as indicated in Table 2.

**Table 2:** The table displays Pearson’s chi-square, likelihood ratio and odd’s ratios (ORs) with corresponding *p*-values for each patient outcome (hospital admission, critical illness, and death) across CRS and ALKA scoring systems.

Patients outcomes	Pearson chi-square, *p*-value		Likelihood ratio, *p*-value		OR (95% CI, *p*-value)	
	CRS	ALKA	CRS	ALKA	CRS	ALKA
Admission	39.47*	78.16*	39.85*	80.32*	1.12 (1.08–1.15)*	3.12 (2.38–4.09)*
Critical illness	3.4	10.21*	3.36	9.77*	2.01 (0.92–4.36)	2.90 (1.31–6.43)*
Death	9.93	13.06*	11.87	14.81*	1.1 (1.05–1.15)	6.25 (1.11–35.25)*

*Bonferroni adjust *p* value <0.01.

### 3.4 Admission

Both the CRS and ALKA scores were significantly associated with hospital admission outcomes. Chi-square analysis revealed a significant association between CRS score and hospital admission (χ^2^ = 39.47, *p* < 0.01) as well as between ALKA score and hospital admission (χ^2^ = 78.16, p < 0.01). The likelihood ratio test also showed a significant association for both ALKA (χ^2^ = 80.32, *p* < 0.01) and CRS scores (χ^2^ = 39.85, *p* < 0.01). In terms of predictive strength, the OR for CRS was 1.12 (95% CI: 1.08–1.15, *p* < 0.001), while the ALKA score had an OR of 3.12 (95% CI: 2.38–4.09, p < 0.001).

### 3.5 Critical illness

ALKA score was significantly associated with critical illness (χ^2^ = 10.21, *p* < 0.01), while the CRS score showed a non-significant association (χ^2^ = 3.4, *p* = 0.18). The likelihood ratio test yielded similar findings, with a non-significant result for the CRS (3.36, *p* = 0.18) and a significant result for the ALKA score (9.77, *p* < 0.01). The OR for ALKA score in predicting critical illness was 2.90 (95% CI: 1.31–6.43). In comparison, CRS had an OR of 2.01 (95% CI: 0.92–4.36), which was not statistically significant (*p* = 0.07).

### 3.6 Death events

Both the CRS and ALKA scores were significantly associated with mortality (CRS: χ^2^ = 9.93, p < 0.01; ALKA: χ^2^ = 13.06, p < 0.01), with likelihood ratio tests confirming these findings (Table 2). The OR for CRS was 1.1 (95% CI: 1.05–1.15), while the OR for ALKA was 6.25 (95% CI: 1.11–35.25). A *post hoc* power analysis showed that the comparison of mortality rates using the ALKA score, with 5.4% mortality in the high-risk group and 0% in the non-high-risk group, had a statistical power of 89.4% at a significance level of 0.05. In contrast, the CRS score comparison, with mortality rates of 6.7 and 2.1% respectively, had a power of 39.8%.

## 4 Discussion

While most patients with COVID-19 experience mild illness, a significant proportion deteriorate and require hospitalisation and intensive care, which underscores the urgent need for a reliable point-of-care clinical assessment tool to enhance patient triage. Such a tool would allow healthcare providers to identify patients who require more intensive intervention in a timely manner, promptly deliver the necessary care, and optimise the allocation of limited healthcare resources, ultimately improving patient outcomes.

Many prognostic scoring systems have been developed to predict disease severity and outcomes in COVID-19 patients (19–30). However, most were designed for hospitalized patients to assist clinical decisions such as ICU admission. For example, the ISARIC 4C Mortality Score, one of the most extensively validated models, performs well in predicting in-hospital mortality (31). Likewise, the CALL score, 4C Deterioration Score, and COVID-GRAM incorporate clinical and laboratory variables to forecast severe disease progression and early deterioration during hospitalization (32–34). While these tools are useful for inpatient management, their utility for early risk stratification at initial presentation remains uncertain and requires further evaluation.

Given these limitations, there is increasing interest in prognostic tools tailored for early triage in the emergency department. Common early warning scores such as NEWS2 and qSOFA have been widely used, but their shortcomings in the COVID-19 context are well documented (27, 28, 34, 35). NEWS2, although commonly applied to detect deterioration in hospitalized patients, lacks specificity for COVID-19 and may lead to over-triage of patients with other acute illnesses (26). Similarly, qSOFA has demonstrated poor predictive accuracy in COVID-19 cases (25). To address this gap, COVID-19 specific scores like PRIEST, RECAP, and CovHos have been introduced to enhance early risk stratification (27–30). The PRIEST score expands on NEWS2 by including demographic data and clinical frailty; however, the subjective nature of frailty assessment limits its broad applicability (30).

Recent systematic reviews and meta-analyses have identified significant limitations in many COVID-19 prediction models. For example, Buttia et al. (7) reviewed 353 prognostic models and reported widespread methodological issues, including high risk of bias, lack of external validation, inadequate handling of missing data, inconsistent definitions of severity and poor generalizability across diverse populations and healthcare environments.

The CRS scoring system, developed and validated by patients in the USA, has been mandated by the Abu Dhabi Department of Health as a risk stratification tool to assess COVID-19 patients and determine their eligibility for treatment with specific COVID-19 medications. Developed from a large cohort of patients in the USA, CRS is an externally validated scoring system, although it has some limitations (5). It utilises 13 distinct risk factors to predict hospital admission and mortality. However, the inclusion of multiple risk factors adds complexity, rendering CRS time-consuming and cumbersome for routine clinical use if the integrated EHR system is not accessible at the time of initial assessment. Moreover, no specific weights have been assigned to the individual risk factors included in CRS, raising concerns regarding the generalisability of CRS validity across diverse patient populations. While the ALKA assessment tool utilises four simple, easily available, initial clinical, and laboratory parameters, it predicts the risk of COVID-19 complications with an accuracy comparable to that of the CRS scoring system. ALKA streamlines the prognostic assessment process of COVID-19 at the time of diagnosis, rendering it more practical for use in busy clinical settings.

This study served as a temporal validation of the ALKA score, originally developed in a UAE cohort, and as a direct comparison with the CRS score, using a new patient group managed during the endemic phase of COVID-19. The ALKA score was applied retrospectively and did not influence clinical decisions, allowing its evaluation under routine care without involvement from the score’s original investigators. The data were collected by a different team than the one that developed the ALKA score, supporting an independent assessment and reducing the risk of bias. Although this was not a multicentre or geographically external validation, it provides meaningful temporal and contextual validation by reflecting a different phase of the pandemic and a distinct operational setting. Both the CRS and ALKA scores were evaluated for their ability to predict hospital admission, progression to critical illness, and mortality. The ALKA score demonstrated prognostic performance that matched or exceeded that of CRS, supporting its potential value as a practical clinical tool.

### 4.1 Performance of CRS and ALKA scoring systems in predicting critical illness, hospital admission, and mortality

For all outcomes (hospital admission, critical illness, and death), ALKA demonstrated a significantly stronger performance than CRS with respect to statistical associations (Pearson’s chi-square, likelihood ratio, and OR).

#### 4.1.1 Hospital admission

Both the ALKA and CRS scores were significantly associated with hospital admission, but ALKA demonstrated superior predictive performance. It showed stronger discriminative ability, higher sensitivity and specificity, and a better overall model fit compared to CRS. The odds ratio for ALKA was notably higher, indicating a stronger relationship with admission outcomes. These results suggest that ALKA may be a more effective tool than CRS for identifying patients who require hospital admission.

#### 4.1.2 Critical illness

Regarding the prediction of critical illness, both the ALKA and CRS scores showed the ability to identify patients at risk, but the difference between the two was not statistically significant. The ALKA score demonstrated better discriminative performance, higher specificity, and explained more variance than CRS, although both models showed good calibration. ALKA was significantly associated with critical illness, with a notably higher odds ratio, while the association with CRS was weaker and did not reach statistical significance. Overall, these findings suggest that ALKA may offer a more reliable prediction of critical illness, but the improvement over CRS was modest.

#### 4.1.3 Mortality

In predicting in-hospital mortality, the ALKA score demonstrated superior performance compared to the CRS score. ALKA achieved excellent discrimination, with an AUC of 0.96 and perfect sensitivity, correctly identifying all patients who died, while maintaining high specificity. The CRS score also showed good discriminative ability but was clearly outperformed across all key metrics. ALKA had a much higher odds ratio and explained substantially more variance, indicating a stronger relationship with mortality outcomes. Both models were well calibrated, but ALKA exhibited a markedly better overall fit. The difference in performance was further supported by a *post hoc* power analysis, which showed that the ALKA score had considerably greater statistical power to detect mortality risk than CRS. These findings suggest that the ALKA score is a more accurate and clinically useful tool for identifying patients at high risk of death.

### 4.2 Implications for clinical practice

CRS, though a useful tool, relies on unquantifiable risk factors that are often obtained from past medical history, which may be incomplete or potentially imprecise. This may introduce inherent inaccuracies in predicting the likelihood of progression to critical illness and contribute to the variability in how these factors are interpreted. Different components of the risk score demonstrate variable predictive abilities for outcomes, suggesting that using uniform weights may compromise the overall accuracy. However, the ALKA score specifically identifies and quantifies early physiological changes that may indicate initial progression toward critical illness, utilising four measurable clinical parameters instead of a broad range of unquantified risk factors. Studies have demonstrated that elevated serum LDH, low serum albumin, and low eGFR at presentation are strong independent predictors of severe disease and critical illness among patients with COVID-19 (36, 37).

In this study, ALKA score outperformed CRS in predicting hospital admissions, progression to critical illness, and mortality. The differences in performance between the two scoring systems can be attributed to their distinct methodologies and clinical variables. The predictive strength of ALKA was demonstrated by its accurate stratification of patients who ultimately succumbed to the disease. The ALKA correctly classified all 8 patients who died as severe cases, whereas the CRS only categorised two of these patients as red (severe), with the remaining six being classified as yellow (moderate). This discrepancy highlights the poor reliability of CRS in accurately identifying COVID-19 patients who are at a high risk of mortality.

While the heterogeneity of a real-world study population of COVID-19 patients enhances the generalisability of our study findings, it may also has affected the performance of risk scores across different patient subgroups (38). Therefore, our study does not fully address how these risk scores may perform across distinct patient subgroups. Future studies should focus on exploring the predictive performance of the ALKA risk score within specific subgroups.

Our study had several limitations. First, as with other retrospective studies, it relied on data extracted from patients’ electronic medical records, which were not originally collected for this purpose. Consequently, some important information may have been missing or incomplete. Second, the study was limited to symptomatic patients attending hospital emergency rooms, which may not accurately represent the broader patient population, particularly those with mild, asymptomatic, and non-hospitalised cases. Third, variability in physician decision-making; based on clinical judgement, radiological findings, and CRS may have led to potential inconsistencies in patient categorisation. Moreover, the ALKA score was developed and validated within a single country, which raises concerns about the external validity of these results. While our findings may be relevant to countries in the Middle East and neighboring Asian regions with comparable demographic and healthcare system characteristics, further validation in varied populations and healthcare settings is required to establish broader applicability. Furthermore, the standardized treatment protocols of the Abu Dhabi Department of Health, including the early administration of monoclonal antibody therapy to all high-risk patients, may have affected both the predictive performance of the risk scores and the observed outcomes by potentially reducing disease progression. Consequently, the influence of these interventions may restrict the generalizability of our findings to healthcare settings where such early treatment approaches are not routinely employed (10).

Despite these considerations, the ALKA score provides a systematic method for risk stratification that can serve as a basis for recalibration or further validation across different populations and healthcare systems. Continuous evaluation and refinement of such models remain important to ensure their clinical relevance amid evolving pandemic dynamics and treatment options. Although CRS has its merits, its complexity hinders its practical use, especially in fast-paced clinical environments where quick decision-making is essential. Additionally, the reliance of CRS on numerous clinical risk factors, which are, at times, unquantifiable, including the requirement for a detailed medical history, coupled with its relatively lower predictive accuracy for key outcomes, such as critical illness, hospital admission, and mortality, makes it less reliable for effectively stratifying patients. In comparison, ALKA scoring system offers a more streamlined approach to risk assessment, rendering it easier to implement in real-world clinical practice.

ALKA, as a risk assessment tool, was developed in response to the COVID-19 pandemic in the UAE and performed well in this study years later, demonstrating its potential as a tool for optimizing healthcare resources and selecting at-risk patients for new therapeutic strategies. Thus, it would be interesting to perform cross-validation of the ALKA COVID-19 critical illness risk assessment in a large multinational dataset, including varied populations and healthcare settings, and to compare them with other clinical risk assessment scoring systems. This would enable a more comprehensive evaluation of the performance of scoring systems across different demographics, potentially resulting in adjustments that enhance their applicability in real-world clinical scenarios. Applying advanced causal inference methods, including target trial emulation, might offer insights into the potential benefits of modifying specific risk factors on improved clinical outcomes in patients with COVID-19 (39–41).

In conclusion, ALKA score demonstrates predictive ability for hospital admission, critical illness, and mortality, rendering it a valuable tool for clinical decision making. In contrast, the intricate nature of CRS makes it onerous and tedious to perform, and the variability in interpreting the risk factors may limit its overall utility. The COVID-19 point-of-care tools would benefit from an external validation study to establish their effectiveness in different healthcare settings.

## Data Availability

The original contributions presented in the study are included in the article/Supplementary material, further inquiries can be directed to the corresponding author.

## References

[1] Bchetnia M Girard C Duchaine C Laprise C. The outbreak of the novel severe acute respiratory syndrome coronavirus 2 (SARS-CoV-2): a review of the current global status. J Infect Public Health. (2020) 13:1601–10. doi: 10.1016/j.jiph.2020.07.011, PMID: 32778421 PMC7402212

[2] Shiels MS Haque AT Berrington de Gonzalez A Freedman ND. Leading causes of death in the US during the COVID-19 pandemic, march 2020 to October 2021. JAMA Intern Med. (2022) 182:883–6. doi: 10.1001/jamainternmed.2022.2476 35788262 PMC9257676

[3] Shaw Stewart PD. Will COVID-19 become mild, like a cold? Epidemiol Infect. (2024) 152:e120. doi: 10.1017/S0950268824001110, PMID: 39370682 PMC11488471

[4] Baj J Karakula-Juchnowicz H Teresinski G Buszewicz G Ciesielka M Sitarz R. COVID-19: specific and non-specific clinical manifestations and symptoms: the current state of knowledge. J Clin Med. (2020) 9:753. doi: 10.3390/jcm9061753, PMID: 32516940 PMC7356953

[5] Halalau A Imam Z Karabon P Mankuzhy N Shaheen A Tu J. External validation of a clinical risk score to predict hospital admission and in-hospital mortality in COVID-19 patients. Ann Med. (2021) 53:78–86. doi: 10.1080/07853890.2020.1828616, PMID: 32997542 PMC7877986

[6] Kompaniyets L Pennington AF Goodman AB Rosenblum HG Belay B Ko JY. Underlying medical conditions and severe illness among 540,667 adults hospitalized with COVID-19, march 2020-march 2021. Prev Chronic Dis. (2021) 18:E66. doi: 10.5888/pcd18.210123 34197283 PMC8269743

[7] Buttia C Llanaj E Raeisi-Dehkordi H Kastrati L Amiri M Mecani R. Prognostic models in COVID-19 infection that predict severity: a systematic review. Eur J Epidemiol. (2023) 38:355–72. doi: 10.1007/s10654-023-00973-x, PMID: 36840867 PMC9958330

[8] Elrobaa IH Khan K Mohamed E. The role of point-of-care testing to improve acute care and health care services. Cureus. (2024) 16:e55315. doi: 10.7759/cureus.55315, PMID: 38434607 PMC10905651

[9] Kurban LAS AlDhaheri S Elkkari A Khashkhusha R AlEissaee S AlZaabi A. Predicting severe disease and critical illness on initial diagnosis of COVID-19: simple triage tools. Front Med. (2022) 9:817549. doi: 10.3389/fmed.2022.817549, PMID: 35223916 PMC8866724

[10] DOH. Use of monoclonal antibodies (Sotrovimab) for treatment of Covid-19. AbuDhabi: Department of Health (2021).

[11] Scharfstein DO Hogan J Herman A. On the prevention and analysis of missing data in randomized clinical trials: the state of the art. J Bone Joint Surg Am (2012);94 Suppl 1:80–84. doi: 10.2106/JBJS.L.00273 22810454 PMC3393113

[12] Peng F Tu L Yang Y Hu P Wang R Hu Q. Management and treatment of COVID-19: the Chinese experience. Can J Cardiol. (2020) 36:915–30. doi: 10.1016/j.cjca.2020.04.010, PMID: 32439306 PMC7162773

[13] Buderer NM. Statistical methodology: I. Incorporating the prevalence of disease into the sample size calculation for sensitivity and specificity. Acad Emerg Med. (1996) 3:895–900. doi: 10.1111/j.1553-2712.1996.tb03538.x, PMID: 8870764

[14] Wynants L Van Calster B Collins GS Riley RD Heinze G Schuit E. Prediction models for diagnosis and prognosis of covid-19: systematic review and critical appraisal. BMJ. (2020) 369:m1328. doi: 10.1136/bmj.m1328 32265220 PMC7222643

[15] Riley RD Snell KIE Archer L Ensor J Debray TPA van Calster B. Evaluation of clinical prediction models (part 3): calculating the sample size required for an external validation study. BMJ. (2024) 384:e074821. doi: 10.1136/bmj-2023-074821, PMID: 38253388 PMC11778934

[16] Riley RD Ensor J Snell KIE Harrell FE Martin GP Reitsma JB. Calculating the sample size required for developing a clinical prediction model. BMJ. (2020) 368:m441. doi: 10.1136/bmj.m441 32188600

[17] DeLong ER DeLong DM Clarke-Pearson DL. Comparing the areas under two or more correlated receiver operating characteristic curves: a nonparametric approach. Biometrics. (1988) 44:837–45. doi: 10.2307/2531595 3203132

[18] Kane S. Post-hoc power calculator (2024). Available online at: https://clincalc.com/stats/power.aspx (Accessed May 31, 2025).

[19] Amezcua-Guerra LM Audelo K Guzman J Santiago D Gonzalez-Flores J Garcia-Avila C. A simple and readily available inflammation-based risk scoring system on admission predicts the need for mechanical ventilation in patients with COVID-19. Inflamm Res. (2021) 70:731–42. doi: 10.1007/s00011-021-01466-x, PMID: 33973018 PMC8109222

[20] Chen Y Zhou X Yan H Huang H Li S Jiang Z. CANPT score: a tool to predict severe COVID-19 on admission. Front Med. (2021) 8:608107. doi: 10.3389/fmed.2021.608107, PMID: 33681245 PMC7930838

[21] Statsenko Y Al Zahmi F Habuza T Gorkom KN Zaki N. Prediction of COVID-19 severity using laboratory findings on admission: informative values, thresholds, ML model performance. BMJ Open. (2021) 11:e044500. doi: 10.1136/bmjopen-2020-044500, PMID: 33637550 PMC7918887

[22] De Vito A Colpani A Saderi L Puci M Zauli B Meloni MC. Is the 4C score still a valid item to predict in-hospital mortality in people with SARS-CoV-2 infections in the omicron variant era? Life. (2023) 13:183. doi: 10.3390/life13010183, PMID: 36676132 PMC9863404

[23] Citu C Citu IM Motoc A Forga M Gorun OM Gorun F. Predictive value of SOFA and qSOFA for in-hospital mortality in COVID-19 patients: a single-center study in Romania. J Pers Med. (2022) 12:878. doi: 10.3390/jpm12060878, PMID: 35743663 PMC9224933

[24] Royal College of Physicians NEWS2 and deterioration in COVID-19 UK. (2025) Available online at: https://www.rcp.ac.uk/news-and-media/news-and-opinion/news2-and-deterioration-in-covid-19 (Accessed April 5, 2025).

[25] Heldt S Neubock M Kainzbauer N Shao G Tschoellitsch T Duenser M. qSOFA score poorly predicts critical progression in COVID-19 patients. Wien Med Wochenschr. (2022) 172:211–9. doi: 10.1007/s10354-021-00856-4, PMID: 34185216 PMC8239483

[26] Williams B. Evaluation of the utility of NEWS2 during the COVID-19 pandemic. Clin Med (Lond). (2022) 22:539–43. doi: 10.7861/clinmed.2022-news-covid, PMID: 36427890 PMC9761427

[27] Espinosa-Gonzalez A Prociuk D Fiorentino F Ramtale C Mi E Mi E. Remote COVID-19 assessment in primary care (RECAP) risk prediction tool: derivation and real-world validation studies. Lancet Digit Health. (2022) 4:e646–56. doi: 10.1016/S2589-7500(22)00123-6, PMID: 35909058 PMC9333950

[28] Salvatore V Trabalza F Casadei L Giostra F. CovHos score for predicting severe respiratory failure in COVID-19 patients presenting at the emergency department. Intern Emerg Med. (2022) 17:1795–801. doi: 10.1007/s11739-022-03006-9, PMID: 35750874 PMC9243846

[29] Goodacre S Thomas B Sutton L Burnsall M Lee E Bradburn M. Derivation and validation of a clinical severity score for acutely ill adults with suspected COVID-19: the PRIEST observational cohort study. PLoS One. (2021) 16:e0245840. doi: 10.1371/journal.pone.0245840, PMID: 33481930 PMC7822515

[30] Thomas B Goodacre S Lee E Sutton L Bursnall M Loban A. Prognostic accuracy of emergency department triage tools for adults with suspected COVID-19: the PRIEST observational cohort study. Emerg Med J. (2021) 38:587–93. doi: 10.1136/emermed-2020-210783, PMID: 34083427 PMC8182747

[31] Knight SR Ho A Pius R Buchan I Carson G Drake TM. Risk stratification of patients admitted to hospital with covid-19 using the ISARIC WHO clinical characterisation protocol: development and validation of the 4C mortality score. BMJ. (2020) 370:m3339. doi: 10.1136/bmj.m3339, PMID: 32907855 PMC7116472

[32] Ji D Zhang D Xu J Chen Z Yang T Zhao P. Prediction for progression risk in patients with COVID-19 pneumonia: the CALL score. Clin Infect Dis. (2020) 71:1393–9. doi: 10.1093/cid/ciaa414, PMID: 32271369 PMC7184473

[33] Liang W Liang H Ou L Chen B Chen A Li C. Development and validation of a clinical risk score to predict the occurrence of critical illness in hospitalized patients with COVID-19. JAMA Intern Med. (2020) 180:1081–9. doi: 10.1001/jamainternmed.2020.2033, PMID: 32396163 PMC7218676

[34] Gupta RK Marks M Samuels THA Luintel A Rampling T Chowdhury H. Systematic evaluation and external validation of 22 prognostic models among hospitalised adults with COVID-19: an observational cohort study. Eur Respir J. (2020) 56:2003498. doi: 10.1183/13993003.03498-2020, PMID: 32978307 PMC7518075

[35] World Health Organization. Clinical management of severe acute respiratory infection when COVID-19 disease is suspected. Geneva: World Health Organization (2020).

[36] Cao A Luo W Wang L Wang J Zhou Y Huang C. The prognostic value of prognostic nutritional index and renal function indicators for mortality prediction in severe COVID-19 elderly patients: a retrospective study. Medicine (Baltimore). (2024) 103:e38213. doi: 10.1097/MD.0000000000038213, PMID: 38758852 PMC11098216

[37] Ozawa T Chubachi S Namkoong H Nemoto S Ikegami R Asakura T. Predicting coronavirus disease 2019 severity using explainable artificial intelligence techniques. Sci Rep. (2025) 15:9459. doi: 10.1038/s41598-025-85733-5, PMID: 40108236 PMC11923144

[38] Guenther B Galizzi MM Sanders JG. Heterogeneity in risk-taking during the COVID-19 pandemic: evidence from the UK lockdown. Front Psychol. (2021) 12:643653. doi: 10.3389/fpsyg.2021.643653, PMID: 33868115 PMC8046913

[39] Xie Y Bowe B Al-Aly Z. Molnupiravir and risk of hospital admission or death in adults with covid-19: emulation of a randomized target trial using electronic health records. BMJ. (2023) 380:e072705. doi: 10.1136/bmj-2022-072705 36882199 PMC9989554

[40] Zuo H Yu L Campbell SM Yamamoto SS Yuan Y. The implementation of target trial emulation for causal inference: a scoping review. J Clin Epidemiol. (2023) 162:29–37. doi: 10.1016/j.jclinepi.2023.08.003, PMID: 37562726

[41] Bhatia A Preiss AJ Xiao X Brannock MD Alexander GC Chew RF. Effect of nirmatrelvir/ritonavir (Paxlovid) on hospitalization among adults with COVID-19: an electronic health record-based target trial emulation from N3C. PLoS Med. (2025) 22:e1004493. doi: 10.1371/journal.pmed.1004493, PMID: 39823513 PMC11790232

