# Supplementary material for: Comparison of the ALKA and CRS scores to predict outcomes among patients with coronavirus disease 2019 infection in United Arab Emirates
Source: Front Med (Lausanne). 2025 Aug 18;12:1553189. doi: 10.3389/fmed.2025.1553189 (PMC12399516; doi:10.3389/fmed.2025.1553189)
Supplement: Supplementary file 1 [file Data_Sheet_1.pdf]

Figure: Flowchart showing the distribution of patients with confirmed COVID-19 infection according to hospital admission, progression to severe and critical illness, and mortality.

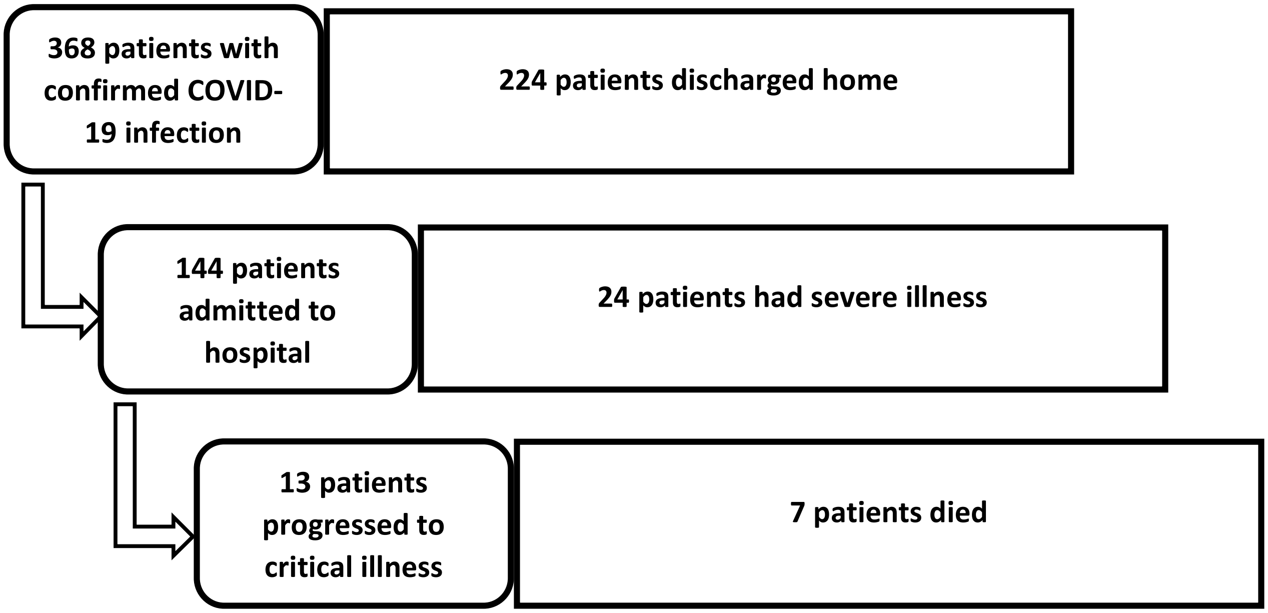

Table: Baseline demographic, clinical and laboratory characteristics of the study cohort stratified by hospital admission status

Characteristic	Total (n=368)	Not Admitted (n=224)	Admitted (n=144)	p-value
**Sex, n (%)**	368			0.17
**Female**	223 (60.6)	142 (63.4)	81 (56.3)	
**Male**	145 (39.4)	82 (36.6)	63 (43.8)	
**Age, years, median (range)**	49 (16–103)	44 (16–83)	60 (17–103)	0.001*
**BMI, kg/m^2, median (range)**	28 (13–53)	29 (15–53)	27 (13–53)	0.033*
**Number of comorbidities, n (%)**				0.001*
**0**	95 (25.8)	75 (33.5)	20 (13.9)	
**1**	106 (28.8)	73 (32.6)	33 (22.9)	
**2**	82 (22.3)	42 (18.8)	40 (27.8)	
**3**	63 (17.1)	27 (12.1)	36 (25.0)	
**≥4**	22 (6.0)	7 (3.1)	15 (10.4)	
**LDH, U/L**	201 (70–799)	192 (70–376)	232 (104–799)	0.001*
**Albumin, g/L**	35 (7–44)	36 (13–44)	32 (7–42)	0.001*
**eGFR, mL/min/1.73m^2**	99 (18–158)	105 (18–145)	87 (5–158)	0.001*

*Statistically significant ($p < 0.05$)

Abbreviations: BMI, body mass index; LDH, lactate dehydrogenase; eGFR, estimated glomerular filtration rate.
