# Supplementary material for: Comparison of the ALKA and CRS scores to predict outcomes among patients with coronavirus disease 2019 infection in United Arab Emirates
Source: Front Med (Lausanne). 2025 Aug 18;12:1553189. doi: 10.3389/fmed.2025.1553189 (PMC12399516; doi:10.3389/fmed.2025.1553189)
Supplement: Supplementary file 2 [file Table_1.docx]

Figure: Flowchart showing the distribution of patients with confirmed COVID-19 infection according to hospital admission, progression to severe and critical illness, and mortality.

**224 patients discharged home**

**368 patients with confirmed COVID-19 infection**

**24 patients had severe illness**

**144 patients admitted to hospital**

**7 patients died**

**13 patients progressed to critical illness**

Table: Baseline demographic, clinical and laboratory characteristics of the study cohort stratified by hospital admission status

| **Characteristic** | **Total**  **(n=368)** | **Not Admitted**  **(n=224)** | **Admitted**  **(n=144)** | **p-value** |
| --- | --- | --- | --- | --- |
| **Sex, n (%)** | 368 |  |  | 0.17 |
| **Female** | 223 (60.6) | 142 (63.4) | 81 (56.3) |  |
| **Male** | 145 (39.4) | 82 (36.6) | 63 (43.8) |  |
| **Age, years, median (range)** | 49 (16–103) | 44 (16–83) | 60 (17–103) | 0.001* |
| **BMI, kg/m^2^, median (range)** | 28 (13–53) | 29 (15–53) | 27 (13–53) | 0.033* |
| **Number of comorbidities, n (%)** |  |  |  | 0.001* |
| **0** | 95 (25.8) | 75 (33.5) | 20 (13.9) |  |
| **1** | 106 (28.8) | 73 (32.6) | 33 (22.9) |  |
| **2** | 82 (22.3) | 42 (18.8) | 40 (27.8) |  |
| **3** | 63 (17.1) | 27 (12.1) | 36 (25.0) |  |
| **≥4** | 22 (6.0) | 7 (3.1) | 15 (10.4) |  |
| **LDH, U/L** | 201 (70–799) | 192 (70–376) | 232 (104–799) | 0.001* |
| **Albumin, g/L** | 35 (7–44) | 36 (13–44) | 32 (7–42) | 0.001* |
| **eGFR, mL/min/1.73m^2^** | 99 (18–158) | 105 (18–145) | 87 (5–158) | 0.001* |

*Statistically significant (p <0.05)

Abbreviations: BMI, body mass index; LDH, lactate dehydrogenase; eGFR, estimated glomerular filtration rate.
